# The influence of training programs on career aspirations: evidence from a cross-sectional study of nursing students in India

**DOI:** 10.1186/s12960-016-0116-9

**Published:** 2016-05-10

**Authors:** Katyayni Seth

**Affiliations:** 91 Church Street, Meerut (250001), India

**Keywords:** Pre-service education, India, Nursing, Labor markets, Training, Employment

## Abstract

**Background:**

Nurses form the largest share of India’s health workforce. This paper explores the relationship between nurses’ pre-service education and labor market aspirations. It investigates supply-side factors shaping students’ career plans and studies the influence that nurse training institutes have on students’ transition into the workforce.

**Methods:**

A cross-sectional survey of 266 nursing students and training administrators at 42 training institutes was conducted in 2014 in two Indian states, Bihar and Gujarat. Piloted questionnaires were used to collect information on the cost and quality of training programs, the background of students, and their career aspirations. Descriptive analyses and multivariate logistic regression analyses were conducted.

**Results:**

A multivariate model on students’ post-graduation plans indicated that students whose institutes provided training in non-technical skills, such as communication and teamwork, were less likely to aim for public sector employment upon completing their training. Similarly, students who joined their training institute because they believed it to be the best place to access job opportunities were less likely to have intentions to seek public sector jobs. Students attending institutes that organized job fairs were also more likely to want to study further or seek private sector employment rather than seeking public sector employment. On the other hand, studying in Bihar and belonging to historically disadvantaged social groups (deemed Scheduled Castes and Scheduled Tribes by the Constitution of India) were factors positively associated with plans to seek public sector employment.

**Conclusions:**

This study helps explain some of the supply-side factors driving the preference for public sector employment among nurses in India by highlighting the influential role of caste, state-level characteristics, and training programs on nursing students’ post-graduation plans. It demonstrates that the strong preference for government jobs among nursing students is linked to the limited role training institutes play in connecting students with other potential employers. In addition, the study indicates that training in non-technical skills, such as communication, makes students more open to pursuing private sector jobs and advanced training programs.

## Background

### Nurses’ training in India

In the past two decades, there has been a tremendous increase in the number of nurse training institutes in India. Between 1997 and 2012, the number of nursing schools and colleges increased by more than 300 %, from 1309 to 5301, resulting in the capacity to train more than 216 000 nurses annually by 2012 [1]. General Nursing and Midwifery (GNM) and Bachelor of Science in Nursing (BSN) are the two main nurse training programs in the country. Between 1997 and 2012, the number of nursing schools offering GNM degrees increased by 277 % and the number of nursing colleges offering BSN rose by 813 %. The number of schools offering training in Auxiliary Nursing and Midwifery (ANM) also increased by 169 % during this period [2]. However, the distribution of nurse training institutes in the country remains geographically skewed. In 2012, the low-income northern states of Bihar and Uttar Pradesh, which have some of the poorest health indicators in the country, had the capacity to produce 7 % of India’s nurses but were home to 25 % of the country’s population [1, 3]. Conversely, the more affluent southern states of Kerala and Karnataka were home to 8 % of India’s population but were producing 27 % of its nurses [1, 3].

Similar to Kenya, South Africa, Thailand, and countries in Latin America and Eastern and Central Europe, private nursing schools in India have grown at an accelerated rate in response to the increased demand for nurses in domestic and foreign labor markets [1, 4, 5]. In 2012, almost 91 % of nursing education in India was being delivered by the private sector [2]. However, ensuring the quality of training at public and private training institutes has been difficult; studies have highlighted significant problems in nursing education in India, including inadequate educational monitoring and governance at the state level and acute faculty and facility shortages [4, 6–9]. In 2004, 61 % of nurse training institutes in India did not meet standards set by the Indian Nursing Council (INC) but continued to function because they were accredited by State Nursing Councils (SNCs) [10]. The INC lacks the legal authority to take action on this since the Constitution of India decrees health to be a state subject and state governments are responsible for implementing health policies. While the INC prepares national guidelines for training programs, prescribes syllabi, and specifies minimum quality criteria for training institutes, state-specific nursing councils are responsible for inspecting and accrediting institutes, conducting examinations, monitoring rules of professional conduct, and maintaining an active register of nursing students and nurses [7, 11].

The increase in nurse training institutes in India has corresponded with an increase in employment opportunities for nursing graduates. It is increasingly recognized that the health needs of India’s rural population are more likely to be met by nurses and paramedics rather than doctors since nurses can provide many clinical and public health services at a lower cost [12]. Nurses are also more likely to work in underserved and rural areas, especially if these are close to their family homes [8]. In addition, compared to physicians, nurses are more willing to work in the public sector because they place a higher value on job security [8]. Studies also suggest that nurses in India prefer working in the public sector because it pays better than most private sector employers [13, 14]. Over half the nurses in India are employed by central and state governments, and opportunities for public sector employment have increased since the launch of the National Rural Health Mission in 2005 [2, 4, 15]. In addition, the Indian Public Health Standards (IPHS), formulated by the central Ministry of Health and Family Welfare (MOHFW) for assessing public health care delivery systems, recommend at least 4 staff nurses (with GNM or BSN qualifications) at primary health centers (PHCs), 10 staff nurses at community health centers (CHCs), 20 staff nurses at sub-district hospitals, 45 to 225 staff nurses at district hospitals, and at least 2 ANMs at a sub-center [16].

The demand for nurses has also increased with the expansion of the domestic private-for-profit health sector in India [1]. The emergence of “five-star” hospitals as destinations for international medical tourism has further increased employment opportunities for qualified nurses in cities like New Delhi [17, 18]. In addition, nursing is seen as a potentially lucrative career because it offers opportunities for migration to higher-income countries [4, 8]. In 2004–2005, India was the leading source of international nurse migrants to Ireland and the UK, and about 10 000 Indian nurses were applying to migrate to the USA with the help of recruiting agencies in New Delhi [11, 19].

Despite the increased demand for nurses, research evidence on their working conditions and job satisfaction shows that nursing in India lacks clear career pathways, in-service training is rare, and pay is low, especially in small private hospitals [6, 13, 14, 20]. In addition, studies conducted in different parts of the country show that private sector health facilities are hiring unqualified or under-qualified personnel to work as nurses in order to reduce their operating costs [8, 13, 14, 21]. Similarly, the public health sector in India is also besieged by shortfalls in the recruitment and deployment of nurses. Hazarika [1] shows that between 2007 and 2009, although there was an increase in the overall stock of nurses, midwives, and doctors in the country, the number of vacant posts in government health facilities for these positions improved little or increased. Budgetary constraints, lack of institutional capacity and transparency in identifying and filling vacancies, legal cases against state health directorates on matters related to the recruitment of health workers, and the unwillingness of many health workers to serve in rural locations have been cited as factors limiting public sector hiring [8, 20, 22]. Furthermore, compared to IPHS recommendations, a sufficient number of public sector positions have not been created for staff nurses. For instance, in 2011, PHCs and CHCs in the states of Bihar and Gujarat were functioning with 21 % and 36 % of the number of staff nurses required, respectively (Table 1). Additionally, in Gujarat, 33 % of positions sanctioned for staff nurses were lying vacant. Apart from their detrimental impact on health care delivery, such gaps in recruitment are a cause of concern because research from other countries suggests that they contribute to migration and unemployment among nurses [23].

**Table 1:** Shortage of staff nurses at PHCs and CHCs in Bihar and Gujarat, 2011

		Gujarat	Bihar
A	Number of functioning PHCs	1 123	1 863
B	Number of functioning CHCs	305	70
C	Staff nurses required^a^	7 542	8 152
D	Sanctioned positions^b^	4 058	1 662
E	Gap (C − D)	3 484	6 490
F	Staff nurses in position	2 705	1 736
G	Vacant positions (D − F)	1 353	−74
H	Shortage (C − F)	4 837	6 416

Source: Rural Health Statistics, 2011 [23]

^a^Required = (4 * A) + (10 * B)

^b^For Bihar, sanctioned data from 2009 is used

Given the rapid increase in training and employment opportunities for Indian nurses over the past two decades, this paper aims to understand the career aspirations of nursing students and the role their background and training programs play in shaping these aspirations. Specifically, it investigates the factors related to the supply of nurses into the labor market which inform students’ intentions to seek employment in the public sector upon completing their studies. This paper contributes to the literature on health worker employment preferences by exploring the influence of training institutes and individual- and state-level characteristics on nursing students’ career plans.

## Methods

### Study setting

Since state-specific policies and institutions regulate nurse training programs in India, this study was conducted in two states in order to understand interstate variations in training programs. The middle-income state of Gujarat and the low-income state of Bihar were selected because of differences in their economic and demographic characteristics and health sector performance (Table 2). Due to its weak public health indicators and infrastructure, Bihar was one of 18 special focus states of the National Rural Health Mission [24]. In 2009–2010, 15 % of children below 2 years of age in the state had not received any vaccinations and only 53 % of deliveries were assisted by trained health personnel. The corresponding figures for Gujarat were 3 % and 85 %, respectively [25]. Relative to Bihar, the private sector in Gujarat plays a more significant role in the provision of health services: in 2008–2009, 56 % of institutional deliveries in Gujarat took place at private facilities, compared to 27 % in Bihar [25]. Out-of-pocket expenditures by households remain the dominant source for health care financing in both states, even though they witnessed a positive and significant rate of growth in public expenditure on health between 2005 and 2011 [26].

**Table 2:** Demographic, socioeconomic, and health indicators for Bihar and Gujarat, for 2011 and in percentages except where indicated

Indicator	Gujarat	Bihar
Population (millions)	60.4	104.1
Population as share of Indian population	5	8.6
Per capita GDP, 2014–2015 (INR)	125 467	39 341
Population below poverty line	16.6	33.7
Rural population	57.4	88.7
Scheduled Caste (SC) population	7	16
Scheduled Tribe (ST) population	15	1
Population density (persons per sq. km)	308	1 106
Literacy rate among males	85.8	71.2
Female literacy rate	63.3	46.4
Sex ratio (females per 1 000 males)	919	918
Female work force participation	23.4	19.1
Infant mortality rate (deaths per 1 000 live births)	36	42
Pregnant women receiving complete antenatal care, 2009	45.7	4.5
Full immunization among children between 12–23 months, 2009	56.6	49.0
Health workers per 1 000 population, 2009	2.89	0.52
Nurses and midwives per 1 000 population, 2009	2.12	0.16

Source: Census 2011 [3], Planning Commission 2013 [35], MOSPI [36, 37], SRS 2014 [38], UNICEF CES-2009 [25], Hazarika 2013 [1]

Bihar faces a severe shortage of health workers. The density of doctors, nurses, and midwives in the state in 2009 was 0.52 per 1000 population [1]. This falls critically below the threshold of at least 2.28 health workers recommended by the World Health Organization in 2006 for high coverage of essential health services [27]. The density of nurses and midwives working in Bihar was 0.16 per 1000 population and the ratio of nurses and midwives per doctor was 0.45, the second lowest among Indian states. On the other hand, in Gujarat, the density of health workers was 2.89 and the density of nurses and midwives was 2.12 per 1000 population; the ratio of nurse and midwives per doctor was 2.77 [1].

### Study design and sample selection

This was a cross-sectional study conducted among nurse training institutes and nursing students enrolled in them. Training institutes were the primary sample units (PSUs), and institutes approved by the INC to admit students into ANM, GNM, or BSN programs for the 2013–2014 academic years were eligible for participation in this study (Table 3). Of the 277 schools and colleges eligible for inclusion, probability-proportional-to-size sampling was used to select 21 institutes in each state. The total sample size of the study was designed to cover 15 % of eligible training institutes and at least five students from each selected institute. The probability of an institute’s selection was proportional to the number of students the INC had approved the institute to admit. At selected institutes, administrators were interviewed by trained surveyors using a piloted questionnaire with close-ended questions. Information was collected on institute finances, infrastructure, training resources, faculty recruitment practices, mobilization of students, placement services, and characteristics of the student and faculty bodies. Participation was voluntary; eight training institutes in Bihar and three in Gujarat refused to participate in the survey, and a second sample was drawn to replace them. Ultimately, all institutes surveyed in Bihar were privately run because sampled public institutes chose not to participate. These refusals could not be replaced with public training institutes due to the low number of such institutes certified by the INC in Bihar.

**Table 3:** Institutes approved by INC to provide training in Bihar and Gujarat, 2013–2014

Training program	Institutes permitted to admit students	Institutes located in the capital district (%)	Public institutes (%)	INC-approved enrolment (seats for 2013–2014)
Bihar				
ANM	43	37	2	1 425
GNM	9	44	0	380
BSN	3	100	0	120
Gujarat				
ANM	83	4	30	2 720
GNM	95	12	27	3 220
BSN	44	16	18	2 020

Overall, 266 students from 42 institutes participated in this study. Convenience sampling at participating institutes was used to select students for inclusion in the survey. From each institute, an average of six registered students present on the day their institute’s administrator was surveyed were asked to fill out a piloted questionnaire with close-ended questions. Trained surveyors selected students by approaching them during a break in classes and explaining the purpose of the survey and the contents of the questionnaire to them. The surveyors distributed questionnaires to students who volunteered to fill them out. Students in the last year of their training program were preferred for inclusion into the study as it was assumed that they would have the most information about their training program and job opportunities related to it. The questionnaires were in Hindi, Gujarati, and English and asked students about their socioeconomic background; educational qualifications; career aspirations; the content, cost, and quality of their training program; and challenges they faced during training and anticipated to face while searching for employment. The questionnaires did not ask for any identifying information and informed consent was obtained from all participating training providers and students.

These surveys were part of a larger study on vocational training undertaken by the World Bank and commissioned by the Government of India. The study design, data collection methods, and questionnaires were approved by the Government before data collection began.

### Quantitative analysis

Two sets of analyses were conducted. First, descriptive analyses were performed to provide insights into training institutes, nursing students, and their post-graduation plans. Second, multivariate logistic regression analysis was used to assess the relationship between multiple student and training characteristics and the dependent variable, students’ intention to seek employment in the public sector upon course completion. Data were cleaned and analyzed with STATA version 13.1 software, and all tests were performed at the 5 % significance level.

## Results

### Characteristics of training institutes

In both Bihar and Gujarat, 17 of the 21 institutes sampled were located in urban areas. Institutes had been operating for a median of 3 years in Bihar and 5 years in Gujarat. Institutes in Gujarat were more likely to be teaching multiple training programs: while 84 % of institutes in Bihar taught only one course, 49 % in Gujarat did the same. The median class size was around 30 in both states, but the average teacher-pupil ratio was better in Gujarat (1:10) compared to Bihar (1:12.5). Administrators reported that among current faculty, teachers in Bihar had a median of 7 years of teaching experience and had never worked in the industry. Similarly, teachers in Gujarat had a median of 6 years of teaching experience, with no other work experience. Although 45 % of institutes in Bihar and 87 % in Gujarat had hired new faculty members in the 3 years preceding the survey, teacher unavailability at private institutes was a cause for concern: 19 % of students in Bihar and 23 % of students at private institutes in Gujarat reported teachers being unavailable when required in the week preceding the survey.

The median course fees charged for an ANM course in Bihar was 72 000 INR or 1.8 times the per capita state gross domestic product (GDP) in 2014–2015. There was a vast difference in fees charged by public and private institutes. In Gujarat, the median course fees for the GNM course was 1000 INR at public institutes and 170 000 INR at private institutes, the latter being 1.4 times the state’s per capita GDP in 2014–2015. Training fees paid by students were the sole source of revenue for 86 % of institutes in Bihar and 21 % of private institutes in Gujarat. Seventy-nine per cent of private institutes in Gujarat also received funds from their parent organizations. Although less than 10 % of private institutes in both states received financial assistance from the state, some private schools (31 % in Bihar and 16 % in Gujarat) received technical assistance in the form of training equipment and training programs for instructors.

About half the surveyed institutes (59 % in Bihar and 41 % in Gujarat) reported undertaking activities to connect students with employers. The most common method was using the personal connections of staff and faculty to help students learn about job opportunities. Only a small group of institutes—around 10 %—participated in formal placement programs such as job fairs.

### Students

In Bihar, almost 90 % of students at INC-approved training institutes were enrolled in ANM training programs, 8 % were training to be GNMs, and less than 5 % were BSN students (Table 4). On the other hand, Gujarat had a higher proportion of GNM students (54 %) compared to ANM (29 %) and BSN students (18 %). Also, in Gujarat, 56 % of the students were studying at public institutes. The median student was younger in Gujarat—20 years compared to 24 years in Bihar. Men were also more likely to be studying nursing in Gujarat: while 96 % of students in Bihar were female, the proportion in Gujarat was 92 %. Relative to the general population of their state, nursing students in Bihar were more likely to be from Scheduled Tribes (8 % compared to 1 % of the state’s population). Similarly, students in Gujarat were more likely to be from Schedule Castes (19 % of students compared to 7 % of the state’s population).

**Table 4:** Sample characteristics of nursing students in Bihar and Gujarat, 2014

Demographic-, socioeconomic-, and training-related characteristics	Gujarat		Bihar		Total	
	*n*	%	*n*	%	*n*	%
	139		127		266	
Age (years)						
<19	35	25.2	8	6.3	43	16.2
19–21	72	51.8	48	37.8	120	45.1
22–24	27	19.4	32	25.2	59	22.2
>24	5	3.6	39	30.7	44	16.5
Gender						
Female	127	91.4	121	95.3	248	93.2
Male	12	8.6	6	4.7	18	6.8
Caste						
Scheduled Caste (SC)	26	18.7	15	11.8	41	15.4
Scheduled Tribe (ST)	24	17.3	10	7.9	34	12.8
Other Backward Caste (OBC)	44	31.7	57	44.9	101	38
General	45	32.4	45	35.4	90	33.8
Marital status						
Not currently married	130	93.5	68	53.5	198	74.4
Household location						
Urban	73	52.5	42	33.1	115	43.2
Rural	66	47.5	85	66.9	151	56.8
Studying in the same district as home district	55	39.6	62	48.8	117	44
Training program						
ANM	40	28.8	112	88.2	152	57.1
GNM	74	53.2	10	7.9	84	31.6
BSN	25	18	5	3.9	30	11.3
Asset possession index						
Lowest	21	15.1	42	33.1	63	23.7
Second	31	22.3	42	33.1	73	27.4
Third	22	15.8	18	14.2	40	15
Fourth	32	23	16	12.6	48	18
Highest	33	23.7	9	7.1	42	15.8
Socioeconomic status						
Lowest	25	18	31	24.4	56	21.1
Second	28	20.1	33	26	61	22.9
Third	32	23	20	15.7	52	19.5
Fourth	24	17.3	32	25.2	56	21.1
Highest	30	21.6	11	8.7	41	15.4

Reflecting the higher level of urbanization in Gujarat, nursing students in the state were more likely to be from urban areas (53 % compared to 33 % of students in Bihar). In both states, only about 2 % of nursing students had prior work experience in any field. Interestingly, marital status among students varied markedly: 7 % of students in Gujarat were married, compared to 47 % in Bihar. With the exception of three students in Bihar, all students were from the state where they were studying. Eighty-two per cent of students in Bihar and 73 % in Gujarat were living in hostels provided by their training institute, and the rest were primarily living with their families.

**Fig. 1:**
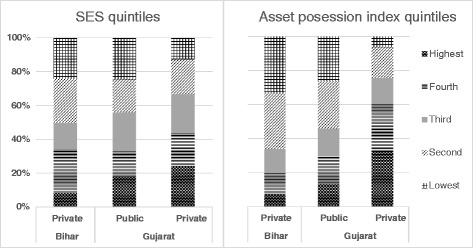
Distribution of students based on socioeconomic status and asset possession indices. Socioeconomic status index components: caste, religion, household (HH) head’s education level and occupation, HH average monthly earnings, structure of the house (pucca/semi pucca/kuccha) and number of rooms, and access to electricity, drinking water, and latrines. Asset possession index components: water pump/tube well/boring, tractor, car/jeep/van, motorcycle/scooter/moped, bicycle, sewing machine, refrigerator, washing machine, computer/laptop, cell phone, television, and radio/transistor

The majority of students at private institutes (80 % in Bihar and 99 % in Gujarat) used personal and family savings to fund their training. Based on the distribution of students across SES quintiles and their position in an asset possession index, students in Bihar came from relatively poorer households. Figure 1 shows that based on these indices, there was also a substantial difference between students attending private and public training institutes in Gujarat. At least 5 % of the students in Bihar and 21 % of the students in Gujarat were from families with incomes less than the average per capita GDP of their state. In Bihar, 7 % of the students belonged to households headed by someone with less than 10 years of education. Thirteen per cent of students in Gujarat belonged to such households.

Students were most likely to have learnt about nurse training programs through friends and family members, followed by media sources such as newspapers. Twenty per cent of students in Bihar and 26 % in Gujarat had enrolled in their training program because their parents or friends told them to do so. Seven per cent of students in Bihar and 24 % in Gujarat knew someone who had studied nursing or midwifery and had advised them to pursue it as a career.

### Labor market aspirations

Gaining employment in the public sector was the primary reason for studying nursing. Ninety per cent of students in Bihar and 63 % in Gujarat wanted a public sector job upon completing training; 10 % of students (5 % in Bihar and 11 % in Gujarat) wanted to work in the private sector, and 20 % (5 % in Bihar and 25 % in Gujarat) wanted to study further. Students reported that the most common way of getting a job was taking exams for public sector employment. When presented with a list of technical and non-technical skills and asked to pick those that are most important for getting a desired job, students ranked working hard and passing these exams the highest. Confidence and luck were also deemed important skills for the labor market. In addition, students anticipated tapping into their personal networks to find employment opportunities: most—55 % in Bihar and 64 % in Gujarat—believed that it was very difficult to get a job without using personal contacts and references. Eleven per cent of students in Bihar and 27 % in Gujarat listed personal contacts as the most popular means of finding employment, and 20 % of students in both states reported institute placement cells as important sources of information about jobs.

Twenty-eight per cent of students in both states regarded the lack of demand for trained nurses as the biggest challenge faced while searching for employment. In Gujarat, a mismatch between skills acquired during training and those demanded by employers was noted as a challenge by 24 % of students. In Bihar, poor communication skills, such as the inability to converse in English, and low confidence were also cited as challenges by 20 % of students. The primary suggestions given by students for improving their training programs were improving placement services and teaching non-technical skills, such as communication and personality development (Table 5).

**Table 5:** Students’ suggestions for improving nurse training institutes in Bihar and Gujarat, 2014, *n* = 266

Student suggestions	Gujarat		Bihar		Total	
	*n*	%	*n*	%	*n*	%
Provide better placements	59	21.2	79	31.5	138	26.1
Provide non-technical/personality development training	69	24.8	58	23.1	127	24.0
Increase exposure to potential employers during training	42	15.1	40	15.9	82	15.5
Provide new tools/equipment	36	12.9	44	17.5	80	15.1
Improve teaching quality	42	15.1	25	10.0	67	12.7
Improve infrastructure	30	10.8	5	2.0	35	6.6

Multivariate logistic regression analysis was conducted to understand factors influencing students’ entry into the labor market. The dependent outcome was plans for public sector employment upon graduation, and independent predictors included student characteristics and features of their training program. Student characteristics comprised of age, gender, marital status, and caste background, along with household-level characteristics such as rural or urban residence and asset ownership. Reasons that prompted students to study nursing were also taken into account as these were hypothesized to influence their labor market decisions as well. Finally, features of their training program and the presence and form of employment assistance offered by their training institutes were included in the model.

Table 6 presents the results of the multivariate analysis, based on which the two strongest predictors associated with plans for public sector employment were joining a training institute because it was believed to be the best place to access job opportunities (*P* = 0.00) and attending a training institute that provided training in non-technical skills (*P* = 0.01). Students who chose to study at their training institute because they believed it to be the best place to access jobs were 1.61 times less likely to have plans to pursue public sector employment, compared to students who joined their training institute for other reasons, after controlling for all other variables. Similarly, students at training institutes that provided training in non-technical skills were 1.20 times less likely to seek public sector jobs compared to students at training institutes that did not provide such training, holding constant all other factors. Conversely, studying in Bihar and belonging to a Scheduled Caste or Scheduled Tribe group was positively associated with intentions to apply for public sector employment (*P* = 0.05), after controlling for other variables. A couple of variables were also significant at the 10 % significance level after controlling for all other independent predictors: students who held the opinion that it is difficult to gain employment without using one’s personal contacts were more likely to have plans to seek public sector employment upon completing their current training (*P* = 0.08). On the other hand, students attending institutes that organized job fairs were significantly less likely to seek public sector employment (*P* = 0.06).

**Table 6:** Multivariate analysis of determinants of intention to seek employment in the public sector (logistic regression)

Predictors	Reference category	*β*	*P*	95 % CI	
				Lower	Upper
Age >21	21 or younger	−0.59	0.20	−1.49	0.30
Male	Female	0.79	0.26	−0.58	2.17
Not currently married	Married	−1.41	0.13	−3.24	0.42
Caste: SC or ST	OBC/general	0.93	0.05	−0.01	1.86
Studying in Bihar	Gujarat	1.67	0.05	0.02	3.31
Household residence: rural	Urban	−0.70	0.11	−1.55	0.15
Household position on asset index		0.14	0.42	−0.20	0.47
Training program: ANM	GNM/BSN	0.48	0.35	−0.52	1.48
Training institute: private	Public	−0.04	0.94	−1.01	0.93
Respondent characteristics					
a)Respondent knew someone who did the same nursing course and recommended nursing as a career		−0.69	0.14	−1.60	0.22
b)Joined their particular nursing institute because it was the best place to get a job		−1.61*	0.00	−2.62	−0.60
c)Think it is important to have personal connections to access jobs		1.32	0.08	−0.17	2.81
Training institute characteristics					
a)Organizes job placements		0.22	0.67	−0.80	1.24
b)Organizes job fairs		−1.47	0.06	−3.02	0.08
c)Informs students about jobs		−0.25	0.60	−1.17	0.67
d)Non-technical skills taught		−1.20*	0.01	−2.11	−0.29
Constant		2.89	0.003	0.98	4.79

*P < 0.05

## Discussion

This study finds that the content and services included in nurse training programs have a strong influence on the career aspirations of nursing students. The results suggest that students graduating from institutes that provide training in non-technical skills are much more likely to pursue further education and jobs in the private sector. In addition, students who chose to attend their training institute because it provides access to job opportunities are less likely to view public sector employment as their immediate career goal. Furthermore, we find that caste plays a significant role in shaping students’ career plans.

This study suffers from limitations that should be avoided in future research. First, it is not able to shed light on the differences between public and private nurse training institutes in Bihar. Relying solely on a sample of private training institutes—although reflective of the actual shares of INC-approved private and public training institutes in the state—could be influencing our findings. This is because private institutes in Bihar, like those in Gujarat, are expected to be younger and more expensive than public institutes. They are also less likely to have the infrastructure and faculty required for teaching GNM and BSN courses. Furthermore, students attending private and public institutes might differ with regard to socioeconomic background and career aspirations. A second limitation of this study is that 80 % of the institutes covered are in urban areas. While this reflects the urban bias in the distribution of nurse training institutes in these two states and the country, the urban location of most of the institutes covered by the survey could be influencing our findings since convenience sampling at sampled institutes was used to invite students to participate in the survey. Third, in order to restrict variations between the types of nurse training institutes surveyed in the two states, this study did not cover institutes accredited solely by State Nursing Councils (SNCs). Although SNCs also approve nursing training institutes, only institutes approved by the INC were included in the sampling frame in order to limit exogenous variations between the two states studied. Further research is needed to understand whether there are significant differences between SNC- and INC-approved training institutes in Bihar and Gujarat. With regard to this study, the absence of SNC-approved institutes from our sampling frame prevents us from providing a comprehensive picture of nurse training programs and students in these states. Lastly, key informant interviews with stakeholders in Bihar, Gujarat, and at the INC on the political economy of nursing education would add richer context to our results. Relatedly, qualitative research, such as focus group discussions and semi-structured interviews with students and nursing graduates, would provide greater insight into the findings.

Despite these limitations, this study adds to the literature on pre-service education for health workers in low- and middle-income countries. Similar to studies from India, Thailand, South Africa, and Kenya, this study also finds a significant difference in fees charged by public and private training institutes in Bihar and Gujarat [4, 28]. In addition, similar to countries like Kenya, there is a predominance of female students at nurse training institutes in the Indian states studied [23]. We find that nursing students are highly unlikely to have ever been employed, and our results match those of other surveys conducted in India that find most students prefer public sector employment [12, 28]. In addition, our findings mirror results from studies of medical students in Timor-Leste that show the important influence of friends and relatives in the choice of profession [29].

Our results also bring to light differences and similarities between nurse training institutes and students in the two states studied. While institutes in Gujarat have multiple sources of revenue, those in Bihar rely heavily on tuition fees. Also, unlike Gujarat, the teacher-pupil ratio at the average training institute in Bihar is above the optimum ratio of 1:10 recommended by the INC. We also find significant demographic and socioeconomic differences between students in the states, which lend credence to formulating state-specific policies on nursing education. For instance, students in Bihar are more likely to be married and from relatively poorer households. With regard to similarities across states, we find that training institutes in both states are quite young. In addition, significant shares of nursing students in both states are from Scheduled Tribe and Caste groups—36 % of students in Gujarat and 20 % in Bihar. Further research is needed to understand the extent to which this is due to the historical origins of the nursing profession in India and due to affirmative action for women and Scheduled Tribe and Caste groups in the recruitment of nurses by the public sector. The extent to which the latter is responsible for the greater likelihood of students from these backgrounds seeking public sector employment upon completing their training also needs to be investigated.

Our findings on students’ post-graduation plans demonstrate the influential role of training providers on students’ educational and career aspirations. We find that enrolling in training institutes that are known to help students access job opportunities reduces the likelihood that a student plans to join the public sector upon completing his or her training. The likelihood of intending to seek employment in the public sector is also lower for students at training institutes that organize job fairs to connect students to potential employers. These results suggest that the strong preference for public sector employment among nursing students could be due to limited career guidance available during training. The process of applying for public sector jobs is relatively transparent compared to finding a job in the private sector, and this could be influencing students’ post-graduation plans. Selection into public sector jobs for nursing is based on state- or district-level competitive examinations and interviews. Vacancies, eligibility criteria, and other details about the recruitment process are announced on publicly available information sources such as newspapers and health department websites. On the other hand, it can be harder to gain information about jobs in the private sector. Since most students have never worked before, they are unlikely to know how to approach private sector employers if they do not have personal contacts working in the same profession and do not attend training institutes that actively connect students to these employers.

This study also finds that nursing students strongly recommend improvements in the placement services currently being provided by their training institutes. However, similar to other vocational training programs in India, this study finds a gap between the education and employment systems for nursing in India [30]. Training institutes do not have substantial incentives to connect students with employers since the INC does not require accredited institutes to provide these services nor does it have any guidelines for training institutes on this topic. Furthermore, in both public and private institutes, the collection of training fees is not linked to labor market outcomes of graduates. In addition, training institutes might lack adequate resources to aid students’ transition into the workforce. This study finds that institutes in Bihar and Gujarat use faculty and personnel connections as the primary means to connect students with jobs. However, since faculty members have not worked in the industry, their understanding of the labor market might be weak and they might not have the skills or time to be effective career counselors. Thus, additional financial investments and human resources may be required to help students. For example, institutes might need to hire people skilled in career counseling, organizing job fairs, and knowledgeable about recruitment practices. The effectiveness and externalities of such investments will need to be considered. For example, if they are financed by an increase in training fees, they could make training unaffordable for current and prospective students from poor backgrounds. In their review of the private sector’s role in the production of nurses in India, Kenya, South Africa, and Thailand, Reynolds et al. [4] find that high tuition fees can result in the inequitable recruitment of students, with wealthier students and those from urban areas being unfairly advantaged. In addition, financial difficulties have been found to cause training disruptions and student attrition at nurse training institutes in Kenya [23].

The influence of training in non-technical skills on students’ labor market aspirations is also an important finding. Our results show that students attending training institutes that provide training in skills such as communication and comportment are more likely to explore further studies and private sector employment as career paths. Empirical literature from the USA and Europe suggests that personality traits and non-cognitive skills, such as time management and the ability to work with others, are important for beneficial labor market outcomes [31, 32]. In their study of Indian firms that hired newly graduated engineers, Blom and Saeki found that employers consider non-technical skills, such as reliability and teamwork, to be more important than engineering-specific technical skills [33]. However, evidence on the labor market impact of training programs providing such non-technical skills to young women is mixed and remains to be studied for the nursing sector in India. A recent impact evaluation in Jordan found that while short-term training in non-technical skills did not have large impacts on generating sustained employment for young, educated women, it resulted in improvements in positive thinking and mental health that might help graduates over a longer period of time [34]. Further research on the demand for non-technical skills by employers of Indian nurses is needed to inform policies on how best to include them in nurse training programs.

Lastly, our results suggest that the labor market’s demand for skilled nurses plays an influential role on the career aspirations of students. We find that there is a greater probability of seeking employment in the private sector or studying further among students in Gujarat. This could be due to the relatively greater number of advanced training institutes in the state (Table 1) and the prominent role of the private sector in the state’s health system. As noted before, it is also possible that the public sector may not be hiring. The main challenges reported by students lend credence to this hypothesis. Conversely, the intentions of students in Bihar to seek jobs in the public sector despite its dismal hiring record could be a result of the limited opportunities for advanced training in the state and the relatively small size of the state’s private health sector. In addition, similar to some private hospitals in states like Delhi and Kerala, private sector hospitals in Bihar may not be hiring qualified nurses [8, 13, 14, 21]. Additional research is needed to investigate whether this is the case.

## Conclusions

This paper has attempted to contribute to a better understanding of the relationship between nurses’ pre-service education and labor market entry. It explored features of nurse training programs and career aspirations of nursing students in two Indian states. In these settings, it is clear that training programs play an influential role in shaping nursing students’ career decisions. This study demonstrates that the strong preference for public sector employment among nursing students is linked to the limited role training institutes play in connecting students with other potential employers. In addition, students’ career goals are influenced by their caste background and the availability of advanced training facilities and private employers in their state.

## Abbreviations

ANM: Auxiliary Nursing and Midwifery
BSN: Bachelor of Science in Nursing
CHC: community health center
GNM: General Nursing and Midwifery
INC: Indian Nursing Council
OBC: Other Backward Castes
PHC: primary health center
SC: Scheduled Caste
ST: Scheduled Tribe
SNC: State Nursing Council
